# Rapeseed oil fortified with micronutrients reduces atherosclerosis risk factors in rats fed a high-fat diet

**DOI:** 10.1186/1476-511X-10-96

**Published:** 2011-06-13

**Authors:** Jiqu Xu, Xiaoqi Zhou, Qianchun Deng, Qingde Huang, Jin'e Yang, Fenghong Huang

**Affiliations:** 1 Oil Crops Research Institute, Chinese Academy of Agricultural Sciences, 2 Xudong Second Road, Wuhan 430062, P.R. China; 2 Department of Nutrition and Food Hygiene, School of Public Health, Tongji Medical College, Huazhong University of Science and Technology, 13 Hangkong Road, Wuhan 430030, P.R. China

**Keywords:** Rapeseed oil, Polyphenols, Tocopherols, Phytosterols, Atherosclerosis, Oxidant stress, Plasma lipids, Inflammation

## Abstract

**Background:**

Micronutrients polyphenols, tocopherols and phytosterols in rapeseed exert potential benefit to cardiovascular system, but most of these micronutrients are removed by the refining process. The aim of this study was to determine the effect of rapeseed oil fortified with these micronutrients on the atherosclerosis risk factors in rats fed a high-fat diet.

**Methods:**

The rodent diet contained 20% fat whose source was refined rapeseed oil (RRO) or fortified refined rapeseed oil with low, middle and high quantities of these micronutrients (L-, M- and H-FRRO). Forty male SD rats were divided into four groups. One group received RRO diet and other groups received L-, M- and H-FRRO diet for 10 weeks.

**Results:**

Micronutrients supplementation significantly increased plasma antioxidant defense capacities, as evaluated by the significant elevation in the activities of GPx, CAT and SOD as well as the level of GSH, and the significant decline in lipid peroxidation. These micronutrients also reduced the plasma contents of TG, TC and LDL-C and increased the ratio of HDL-C/LDL-C. In addition, in parallel with the enhancement of these micronutrients, plasma levels of IL-6 and CRP declined remarkably.

**Conclusion:**

Rapeseed oil fortified with micronutrients polyphenols, tocopherols and phytosterols may contribute to prevent atherogenesis by ameliorating plasma oxidative stress, lipid profile and inflammation.

## 1. Introduction

Cardiovascular disease (CVD) is the largest cause of premature death in most developed and developing countries and it is also an increasingly important source of disability and contributes in large part to the escalating costs of health care. Atherosclerosis is the most common pathologic process underlying CVD. It is clear that several risk factors such as oxidant stress [1], lipid abnormalities [2] as well as chronic inflammation [3] have been correlated to both the initiation and the progression of atherosclerosis and subsequent CVD.

Rapeseed oil is one of the major and cheapest vegetable oils for human diet in china and many other countries. This kind of plant oil has the lowest concentration of saturated fatty acids in all commonly consumed oils and high level of monounsaturated fatty acids [4]. In addition, it is also the major source of linoleic acid and α-linolenic acid, and it is, so far, the closest to the optimum to meet the basic requirements of essential fatty acids in the body [5]. A lot of studies have shown that rapeseed oil can reduce serum total cholesterol (TC) and/or low-density lipoprotein cholesterol (LDL-C) when fed in place of saturated fatty acids [6-8], which means that rapeseed oil possesses significant health benefits in reducing atherosclerosis risk. In addition to triacylglycerols, rapeseed oil also contains many micronutrients such as tocopherols, phytosterols and phenolic compounds which have been reported to impart health benefits or desirable physiological effects. For example, these micronutrients are the most important natural antioxidants which act as free radical scavengers or complexers of prooxidant metals, and the various bioavailable antioxidants present in rapeseed oil work in concert to upgrade the complex antioxidant network which increase antioxidant capacity higher than that provided by each separate compound [9-11]. Phytosterols have been reported to exert hypocholesterolemic effect by inhibiting cholesterol absorption [12]. Studies also point out an independent effect of phenolics improving plasma lipid profiles [13,14]. In addition, all these compounds are also believed to have antiinflammatory effects [14-16]. All these beneficial effects of these micronutrients might participate in preventing the initiation and development of atherosclerosis. However, although most unwanted components are removed, the traditional processing technology currently used in the world (extraction and refining) markedly decreases the content of these micronutrients, which will have an adverse effect on the protection of cardiovascular system.

In order to increase the levels of these micronutrients in rapeseed oil, some improving processing technologies [17] which can preserve the content of these micronutrients in rapeseed oil have been researched, and artificially adding micronutrients to refined oil may be another simple and expedient method. The present study tried to investigate that whether rapeseed oil fortified with micronutrients (polyphenols, tocopherols and phytosterols) can reduce atherosclerosis risk factors in rats fed a high-fat diet.

## 2. Materials and methods

### 2.1 Fortification of rapeseed oil with micronutrients

The refined rapeseed oil was obtained from Hulunbuir Jinjiao Bio-chemical Ltd, and its levels of tocopherols, phytosterols and phenolic compounds were analyzed and shown in table 1. Tocopherols (wuhan yuancheng, china), fat-soluble polyphenols (pulimeidi biotech, china) and plant sterol esters (Vegapure 95E^®^, Cognis GmbH, Germany) were used as the fortificants in this study. The rapeseed oil was fortified with low, middle and high contents of these fortificants and the contents of these micronutrients in the fortified oil were also shown in table 1.

**Table 1:** Micronutrients contents in different rapeseed oils.

mg/kg oil	RRO	L-FRRO	M-FRRO	H-FRRO
polyphenols	36.7	200	400	800
phytosterols	9035.8	10000	20000	40000
tocopherols	143.8	500	1000	2000

### 2.2 Animals and diets

Forty male Sprague-Dawley rats, initially weighing 150-170 g, were purchased from Sino-British Sippr/BK (Shanghai, China). The rats were housed individually and maintained at a controlled ambient temperature (24 ± 1 °C) under diurnal conditions (light-dark: 08:00-20:00) with access to laboratory chow and tap water ad libitum. After the rats were acclimated for 1 week, animals were randomly divided into four groups of 10 animals each, consisting of the refined rapeseed oil group (RRO), fortified refined rapeseed oil with low, middle and high contents of these fortificants (L-, M-, and H-FRRO) groups. The high-fat diet contained 20% casein, 35% maize starch, 15% glucose, 5% cellulose, 3.5% mineral mixture (AIN-93M), 1% vitamin mixture (AIN-93M), 0.2% choline bitartrate, 0.3% DL-methionine and 20% fat. The fat in the diet was provided by different rapeseed oils mentioned above. All animals were weighed twice a week and food intake was measured weekly. The animals were cared for in accordance with *the Guiding Principles in the Care and Use of Animals*. The experiment was approved by the Oil Crops Research Institute Council on Animal Care Committee, Chinese Academy of Agricultural Sciences.

### 2.3 Blood processing

After 10 weeks of treatment, rats were fasted for 16 hours and then killed under anaesthesia, blood was collected in the presence of sodium heparin from the heart immediately. Blood samples were centrifuged at 1500 g (10 min, 4°C) and the plasma was stored at −80°C until analysis.

### 2.4 Plasma lipids analysis

The plasma concentrations of triglyeride (TG), TC, LDL-C and high-density lipoprotein cholesterol (HDL-C) were measured with commercial kits (Zhongsheng Beikong Biotech Company, China).

### 2.5 Assay of plasma antioxidant capacity and lipid peroxidation

#### 2.5.1 Determination of glutathione peroxidase (GPx)

GPx activity was determined by the use of commercial kit (Nanjing Jiancheng Bioengineering Institute, China) based on the method of Sazuka et al. [18]. Briefly, plasma mixed with GSH and hydrogen peroxide was incubated at 37 °C for 3 min, followed by the addition of 10% trichloro acetic acid. After centrifugation, the supernatant was collected and mixed with disodium hydrogen phosphate and 5,5,-dithiobis(2-nitro-benzoic acid). The absorbance was recorded at 412 nm. The unit of GPx activity was expressed as micromoles GSH oxidation per 5 minute per 0.1 millilitre plasma.

#### 2.5.2 Determination of catalase (CAT)

CAT activity was estimated according to the method of Goth [19] with slight modification. Briefly, 50 μl of sample was mixed with 50 μl of substrate (6.5 μM hydrogen peroxide in phosphate buffer) for 60 s, then 100 μl of 32.4 mM ammonium molybdate solution were added and absorbance change was measured at 405 nm. One unit of the enzyme was defined as millimoles of hydrogen peroxide degraded per minute per millilitre plasma.

#### 2.5.3 Determination of superoxide dismutase (SOD)

SOD activity was estimated basing on the method of Kono [20] with slight modification. Briefly, the reaction was initiated by mixing an appropriate plasma with 0.5 mM hypoxanthine, 0.5 mM hydroxylamine and 0.01 U xanthine oxidase in the buffer, containing of 104 mM potassium phosphate, 78 mM sodium borate and 0.025 mM EDTA (PH 7.0) at 37 °C for 30 min in a reaction volume of 100 μl. The reaction was terminated by adding 0.2 ml of 16% (v/v) acetic acid solution containing 2.6 mM sulfanilic acid and 38.6 μM naphthyl ethylenediamine and the absorbance at 550 nm was recorded for the calculation of SOD activity. Under the conditions, one nitroso unit of enzyme activity was calculated as that inhibiting 50% of the oxidation of hydroxylamine without an enzyme source.

#### 2.5.4 Determination of reduced glutathione (GSH)

The GSH content was estimated by the use of commercial kit (Nanjing Jiancheng Bioengineering Institute, China) based on the method of Moron et al. [21]. Briefly, the plasma was precipitated with 50% trichloro acetic acid and then centrifuged at 1000 g for 5 min. The reaction mixture containing 50 μl of supernatant, 200 μl of 0.2 M Tris-EDTA buffer (PH 8.9) and 10 μl of 0.01 M 5,5,-dithiobis(2-nitro-benzoic acid) was kept at room temperature for 5 min, and then measured at 412 nm. The GSH concentration was calculated using a GSH standard curve.

#### 2.5.5 Determination of total antioxidant capability (T-AOC)

The T-AOC was assayed with commercial kits (Nanjing Jiancheng Bioengineering Institute, China).

#### 2.5.6 Determination of thiobarbituric acid reactive substances (TBARS)

TBARS level was measured by the method of Buege and Aust [22]. Briefly, the plasma was incubated reagent containing 0.375% thiobarbituric acid, 15% trichloro acetic acid, 0.25 M HCl, and 6.8 mM 2,6-di-tert-butyl-4-methylphenol for 60 min in a boiling water bath. The mixture was centrifuged at 3000 rpm for 15 min, the absorbance of the supernatant was recorded at 532 nm by using 1,1,3,3-tetraethoxypropane as standard. The lipid peroxidation was expressed as TBARS in nanomoles per millilitre plasma.

### 2.6. Assay of plasma inflammatory markers

Plasma interleukin 6 (IL-6) and C-reactive protein (CRP) levels were determined using rat IL-6 ELISA kit (eBioscience San Diego, CA) and rat CRP ELISA kit (eBioscience San Diego, CA), respectively. All the conditions and procedures were consistent with the instructions of these kits.

### 2.6 Statistical analyses

Values are presented as mean ± SD. The data were analyzed by one-way ANOVA, followed by the Fisher PLSD post hoc test if the overall differences were significant (*p*< 0.05). All statistical analyses were performed using SPSS 13.0 statistical software (SPSS Inc., Chicago, IL) and a difference was considered significant when *p *< 0.05.

## 3. Results

### 3.1. Food intake and food utilization rate

There were no remarkable differences in food intake or food utilization rate were observed among all groups throughout the experiment. The average food utilization rate (%) was 15.31 ± 2.35, 15.83 ± 3.22, 14.85 ± 2.40, 15.04 ± 2.20 for RRO group, L-, M-, and H-FRRO groups, respectively.

### 3.2. Plasma antioxidative capacity and lipid peroxidation

**Figure 1:**
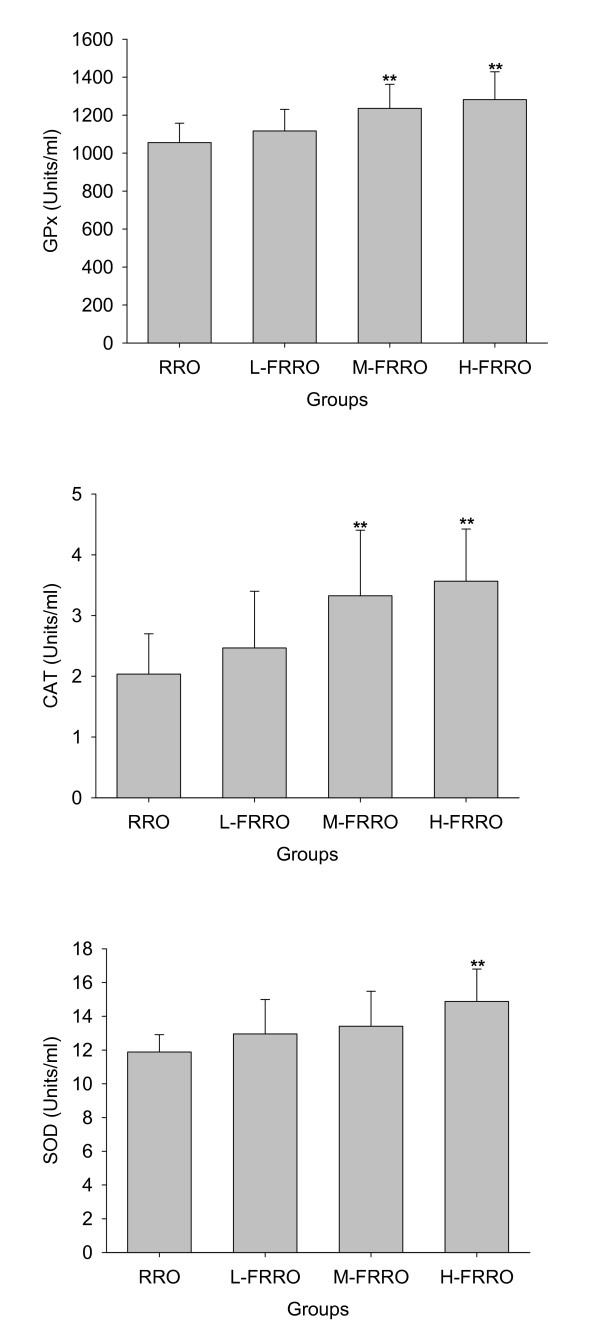
**Effects of rapeseed oil fortified with micronutrients (polyphenols, tocopherols and phytosterols) on the activities of antioxidant enzymes (GPx, CAT and SOD) in plasma of rats fed a high-fat diet**. RRO: the refined rapeseed oil group; L-. M- and H- FRRO: fortified refined rapeseed oil with low, middle and high contents of micronutrients groups. Bars represent the mean ± SD from 10 animals in each group. ** *p *< 0.01 compared to the RRO group.

**Figure 2:**
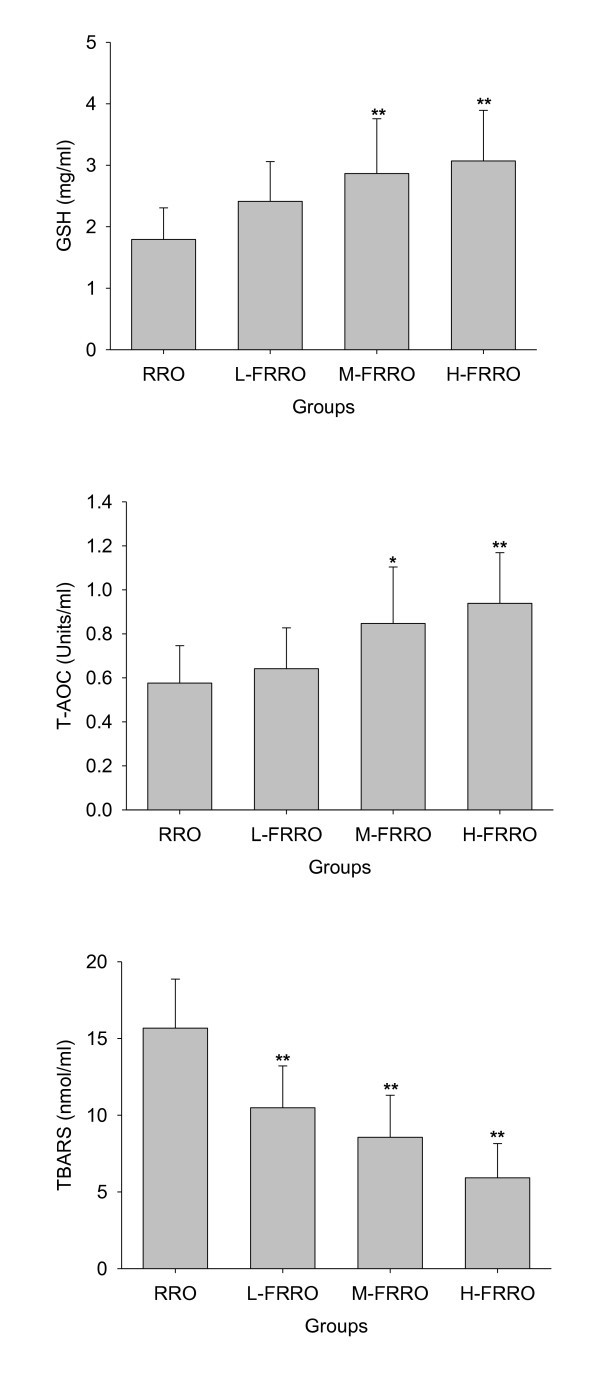
**Effects of rapeseed oil fortified with micronutrients (polyphenols, tocopherols and phytosterols) on the levels of GSH, T-AOC and contents of TBARS in plasma of rats fed a high-fat diet**. RRO: the refined rapeseed oil group; L-. M- and H- FRRO: fortified refined rapeseed oil with low, middle and high contents of micronutrients groups. Bars represent the mean ± SD from 10 animals in each group. * *p *< 0.05 and ** *p *< 0.01 compared to the RRO group.

As can be seen in Figure 1 and 2, significant increases in the activities of plasma antioxidant enzymes GPx and CAT in M- and H-FRRO groups and SOD in H-FRRO group were observed when compared with the RRO group. Animals in M- and H-FRRO groups revealed significantly higher plasma GSH levels than their counterparts in RRO group. As the overall antioxidative capacity, plasma T-AOC in M- and H-FRRO groups was significantly higher than that in RRO group. In addition, when plasma TBARS were examined as the marker of lipid peroxidation, animals in all FRRO groups revealed marked lower TBARS levels than that in RRO group.

### 3.3. Plasma lipids

**Figure 3:**
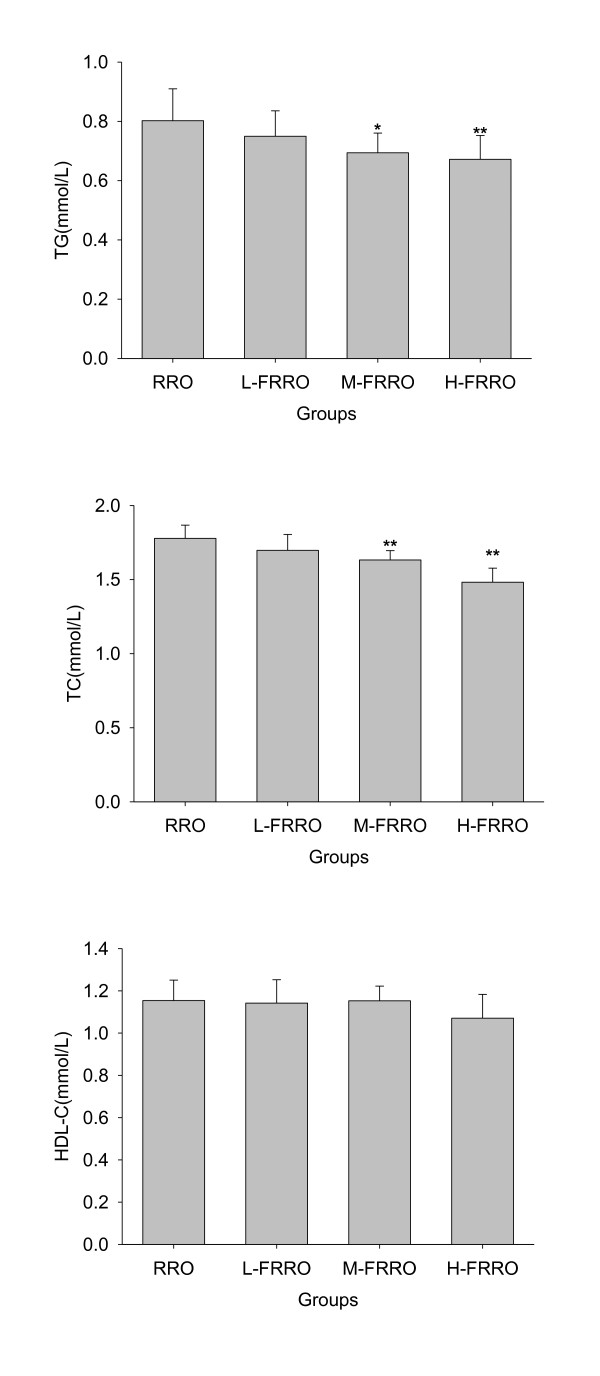
**Effects of rapeseed oil fortified with micronutrients (polyphenols, tocopherols and phytosterols) on TG, TC and HDL-C in plasma of rats fed a high-fat diet**. RRO: the refined rapeseed oil group; L-. M- and H- FRRO: fortified refined rapeseed oil with low, middle and high contents of micronutrients groups. Bars represent the mean ± SD from 10 animals in each group. * *p *< 0.05 and ** *p *< 0.01 compared to the RRO group.

**Figure 4:**
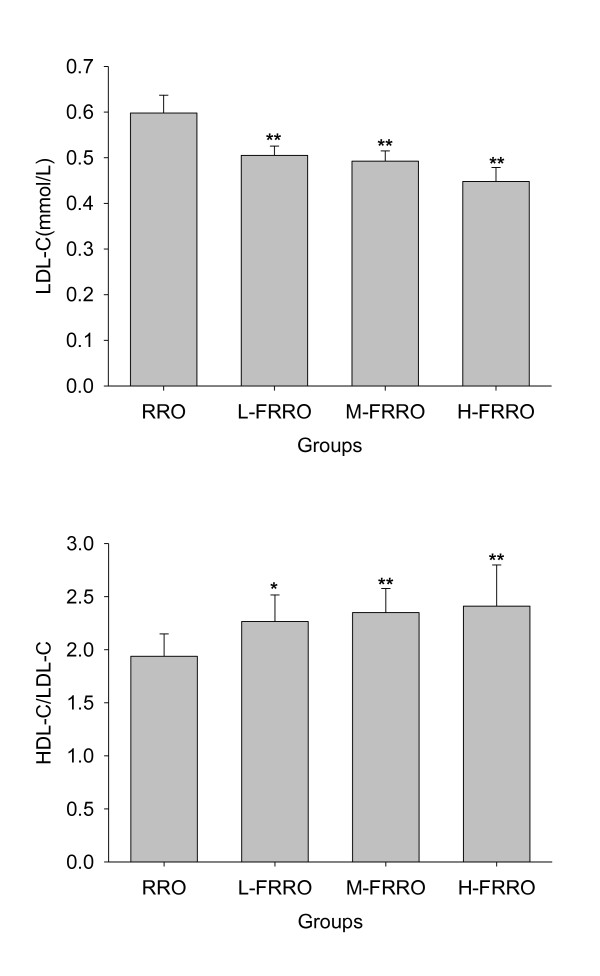
**Effects of rapeseed oil fortified with micronutrients (polyphenols, tocopherols and phytosterols) on plasma LDL-C and on the ratios of plasma HDL-C/LDL-C of rats fed a high-fat diet**. RRO: the refined rapeseed oil group; L-. M- and H- FRRO: fortified refined rapeseed oil with low, middle and high contents of micronutrients groups. Bars represent the mean ± SD from 10 animals in each group. * *p *< 0.05 and ** *p *< 0.01 compared to the RRO group.

As shown in Figure 3 and 4, there were marked decline in the levels of both plasma TG and TC in M- and H-FRRO groups when compared with those in RRO group. Because animals in all FRRO groups showed significantly lower LDL-C levels than rats in RRO group and HDL-C levels were similar in all groups, all FRRO groups were therefore had the significantly higher ratios of HDL to LDL cholesterol than RRO group.

### 3.4. Plasma inflammatory

**Figure 5:**
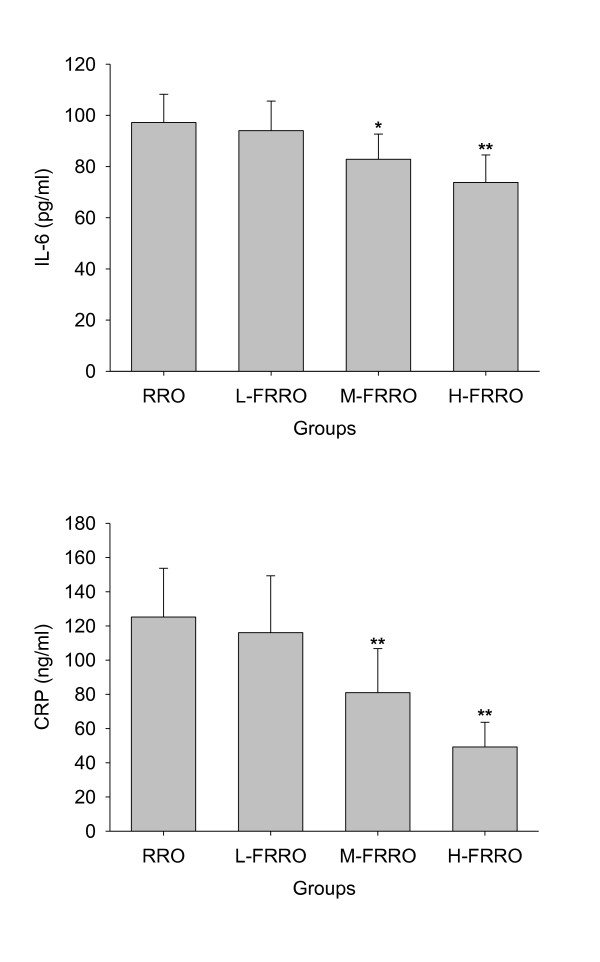
**Effects of rapeseed oil fortified with micronutrients (polyphenols, tocopherols and phytosterols) on the level of IL-6 and CRP in plasma of rats fed a high-fat diet**. RRO: the refined rapeseed oil group; L-. M- and H- FRRO: fortified refined rapeseed oil with low, middle and high contents of micronutrients groups. Bars represent the mean ± SD from 10 animals in each group. * *p *< 0.05 and ** *p *< 0.01 compared to the RRO group.

The effect of FRRO on plasma IL-6 and CRP levels are shown in Figure 5. The FRRO decreased the plasma IL-6 and CRP levels in a dose-dependent manner, and both plasma IL-6 and CRP levels in rats of M- and H-FRRO groups were significantly lower than that of RRO group.

## 4. Discussion

As one of the commonly consumed vegetable oils in the world, rapeseed oil has the potential to meet consumers' dietary needs because it has the lowest concentration of saturated fatty acids, a very favorable ratio of linoleic acid to α-linolenic acid and the predominantly of monounsaturated fatty acids. The optimum fatty acid composition of this oil can exert a hypo chotesterolsemic effect [6-8,23,24] when fed in place of higher saturated fatty acids-containing fats and thus benefits the protection of CVD.

In recent years, an increasing interesting has been taken in the study of many micronutrients contained in rapeseed because of their excellent bioactivity. These micronutrients include phytosterols and various antioxidants such as polyphenols, coenzyme Q and tocopherols, and the joint actions of all these bioactive molecules may contribute to prevent the risk of atherosclerosis. Unfortunately, most of these micronutrients are lost during the oilseed oil refining. The present study examined the effect of reducing atherosclerosis risk factors by artificially adding micronutrients (polyphenols, tocopherols and phytosterols) to refined rapeseed oil.

The imbalance between the cellular free radical formation and the antioxidant defense leads to oxidative stress. The relative excessive production of free radicals can attack many different cellular components, including lipids, proteins and DNA, which initiates the processes of atherogenesis through cell dysfunction [25]. In fact, oxidative stress is the unifying mechanism for many CVD risk factors [26]. For example, free radicals mediate many signaling pathways which underlie vascular inflammation in atherogenesis [27]. However, the deleterious effects of oxidative stress can be prevented by enzymatic and non-enzymatic antioxidant defense mechanism. In mammals, the most important antioxidant enzymes include SOD which converts superoxide to hydrogen peroxide, GPx and CAT which are responsible for converting hydrogen peroxide to water [28]. As a very important non-enzymatic antioxidant, GSH can react directly with free radical or act an electron donor in the reduction of peroxides catalyzed by GPx [29]. In addition to scavenge free radicals directly, supplement of these micronutrients in the present study also significantly elevated plasma activities of SOD, CAT and GPx as well as level of GSH, which led to the remarkable increase of T-AOC and were thus favorable to attenuate oxidative stress. As a result, plasma lipid peroxidation levels were significantly decreased with the supplement of these micronutrients. In accord with these findings, these micronutrients naturally abundant in rapeseed oil have been reported to increase antioxidant status and lipid peroxidation in plasma [17] as well as in brain [30].

Hyperlipemia is also well known to be closely linked to arteriosclerosis and other cardiovascular disease. It has been reported that rapeseed oil possessed hypolipidemic properties [7,31] and which could be due to its optimum fatty acid composition. In line with expectations, the further reduction in plasma TG, TC and LDL-C were observed with the fortification of rapeseed oil with micronutrients in this study. The micronutrients phytosterols and polyphenols were both responsible for these beneficial changes. Many studies have shown that intake of phytosterols is effective at lowering plasma TC and LDL-C. Phytosterols have a similar chemical structure with cholesterol but themselves are absorbed only in trace amounts [32], thus they inhibit cholesterol absorption including recirculating endogenous biliary cholesterol which is a key step in cholesterol elimination [32]. These meant that although rodent diet contained little cholesterol in this study, inhibition of intestinal cholesterol absorption was still the main mechanism responsible for the cholesterol-lowering effect of phytosterols. Besides, the hypolipidaemic effect of the phytosterols were also associated with the down-regulation of hepatic acyl CoA:cholesterol acytransferase activity [24] and the increasing LDL receptor expression [33]. Polyphenols have been shown to reduce plasma TG, TC and LDL-C by altering hepatic triglyceride assembly and secretion, cholesterol absorption and the processing of lipoproteins in plasma [14]. Although these micronutrients did not affect plasma HDL-C level in the present study, the increase in the ratio of HDL to LDL cholesterol still meant that FRRO had a significant protective effect with regard to atherosclerosis. It should be pointed out that although rat was used as a model for lipid metabolism in our investigation and others [17,34], the plasma lipid and lipoprotein profile in this species are very different from human, and therefore further experimentation is needed to verify the beneficial effects on lipid metabolism of human subjects.

Recent advances in both the basic and clinical science have recognized the critical role of inflammation in all stages of atherosclerosis [35-37]. Various proinflammatory risk factors (oxidized LDL, infectious agents, etc) can trigger the production of proinflammatory cytokines which contribute to development and progression of atherosclerosis. IL-6 and CRP are two of the most important proinflammatory cytokines, and both of which have been served as inflammatory markers for evaluation of atherosclerotic risk [38-40]. In parallel with the enhancement of these micronutrients in the present study, plasma levels of both IL-6 and CRP declined remarkably, which meant that FRRO was able to improve inflammation status. Each ingredient of these micronutrients has been suggested to play a role in inhibition of inflammatory cytokine production [14-16]. For example, both polyphenols and tocopherols have been shown to retard LDL oxidation through their antioxidant property [15,41] and inhibit the synthesis of leukotriene B_4 _(LTB_4_) [42,43]. Polyphenols can also exert the anti-inflammatory effect by inactivating STAT1 and NF-κB [44].

In conclusion, rapeseed oil fortified with tocopherols, polyphenols and phytosterols has the ability to ameliorate oxidative stress, lipid profile and inflammation of plasma. These results suggested that the rapeseed oil fortified with these micronutrients might contribute to prevent atherogenesis and then reduce the incidence of CVD.

## Abbreviations

CVD: cardiovascular disease; TG: total triglyceride; TC: total cholesterol; LDL-C: low-density lipoprotein cholesterol; HDL-C: high-density lipoprotein cholesterol; RRO: refined rapeseed oil; FRRO: fortified refined rapeseed oil; GPx: glutathione peroxidase; CAT: catalase; SOD: superoxide dismutase; GSH: glutathione; T-AOC: total antioxidant capability; TBARS: thiobarbituric acid reactive substances; IL-6: interleukin 6; CRP: C-reactive protein

## Competing interests

The authors declare that they have no competing interests.

## Authors' contributions

JX designed and wrote a first draft of the paper. XZ,QD and JY carried out all the experiments. QH performed the data analysis and created the figures. FH contributed to the design of the study, reviewed the manuscript and contributed to the final version. All authors contributed to and have approved the final manuscript.
